# Atypical Periprosthetic Femoral Fracture Associated With Prolonged Bisphosphonate Therapy: A Two-Centered Retrospective Case Series and Literature Review

**DOI:** 10.7759/cureus.24514

**Published:** 2022-04-26

**Authors:** Konlawat Sabsuantang, Thanainit Chotanaphuti, Siwadol Wongsak, Paphon Sa-Ngasoongsong, Saradej Khuangsirikul, Kulapat Chulsomlee, Noratep Kulachote

**Affiliations:** 1 Orthopedics, Chakri Naruebodindra Medical Institute, Faculty of Medicine Ramathibodi Hospital, Mahidol University, Samut Prakan, THA; 2 Orthopedics, Phramongkutklao Hospital and College of Medicine, Bangkok, THA; 3 Orthopedics, Faculty of Medicine Ramathibodi Hospital, Mahidol University, Bangkok, THA

**Keywords:** implant failure, reoperation, complication, bisphosphonate, osteoporosis, hip arthroplasty, atypical periprosthetic femoral fracture

## Abstract

To date, atypical periprosthetic femoral fractures (APFFs) are not well-understood due to a relatively small number of studies on the topic. Moreover, there is a paucity of guidance regarding the appropriate treatment strategy. We would therefore like to present and analyze a series of five APFF cases (one incomplete APFF and four complete APFFs) that were treated in two university hospitals; a review of the literature is also provided. The results provide additional and useful information regarding the treatment strategy for APFFs.

## Introduction

Bisphosphonates (BPs) are one of the most commonly used osteoporotic drugs because of their proven efficacy to reduce the incidence of fragility fractures [1]. However, long-term use of BPs can cause a condition called severe suppression of bone turnover (SSBT), resulting in complications such as atypical femoral fractures (AFFs). The American Society of Bone and Mineral Research (ASMBR) Task Force reviewed and proposed revisions to AFFs' case definition in 2014 comprising clinical details, specific fracture characteristics, and expected treatment outcomes [2], but did not include atypical periprosthetic femoral fractures (APFFs). APFFs are fractures with atypical features occurring around the femoral prosthesis of hip arthroplasty. Because of the rarity of cases and limited awareness of this condition, to date, there are far fewer APFF studies than those on AFFs and typical periprosthetic femoral fractures (PFFs). As a result, APFFs are still poorly understood, particularly the treatment outcomes and effective treatment strategy.

Theoretically, APFFs seem to share some patient characteristics, risk factors, and difficulties in treatment with AFFs and PFFs. However, the treatment for APFFs can be more complex, because they combine the challenging poor healing potential of AFFs and the fixation difficulty due to the presence of femoral stem prosthesis in PFFs. To achieve fracture healing in AFFs, the recommendations require not only using intramedullary nail (IMN), which provides a mechanical advantage and is less disturbed from osteoclast inactivation, but also considering biological augmentations in the selected patient [3]. Plating in AFFs also results in a higher failure rate compared to IMN (31.3% to 12.9%, respectively) [4]. In APFFs, IMN cannot be used, and the fixation must be a plate, which is generally more difficult and especially so in proximal fixations. Previous evidence showed a higher rate of fracture-related complications in APFFs compared to AFFs [5] and PFFs [6,7]. Therefore, the effective fixation strategy for APFFs should be explored. Moreover, since they can, just like AFFs, be occult or incomplete fractures, the appropriate decision-making for conservative and operative treatment must be considered for APFFs.

This study aims to (1) report on the patient characteristics, treatment methods, and outcomes of the selected APFF cases; (2) analyze them in terms of potential causes for success or failure; and (3) review the literature related to APFF treatment outcomes and develop an up-to-date treatment strategy.

## Case presentation

This study was approved by the Institutional Review Board of the Faculty of Medicine, Ramathibodi Hospital, Mahidol University (COA. MURA 2019/760). We retrospectively reviewed the patients with PFFs operated on in 2006-2021 at Ramathibodi Hospital and Phramongkutklao Hospital. Inclusion criteria were the patients who (1) were diagnosed with PFFs around the femoral stem and (2) had at least four of the five major features listed in the ASBMR 2014 revised case definition [2]. Patients with pathological fractures from tumors were excluded. 

A total of five cases, one incomplete and four complete fractures, were included in this study. All cases involved female patients with an average age of 66 years. Four (80%) had underlying autoimmune diseases and received prednisolone and immunosuppressive medications. All fractures occurred at or around the mid or tip of stem (Vancouver B1), and all prostheses had a well-fitted cemented femoral stem. The average duration of bisphosphonates was 10.6 years (range 3-14 years). The mean duration of prodromal symptoms was 3.4 months (range 1-8 months). One incomplete APFF, with a previous unipolar hip prosthesis, was treated with revision hip arthroplasty due to failed conservative treatment. In the four complete APFFs, one was treated with revision long femoral stem with plate augmentation, and the other three were treated with open reduction with internal fixation (ORIF) with plate and screws using additional augmentation techniques. Three of five fractures (one incomplete and two complete) achieved union with a mean union time of six months after the first operation. The residual two fractures were re-operated with fixation and finally achieved fracture union. The data from each case are presented separately and then summarized in Table 1.

**Table 1:** Summary of the patients’ characteristics and treatment in this study F: female; Y: yes; RA: rheumatoid arthritis; SLE: systemic lupus erythematosus; CVA: cerebrovascular accident; DLP: dyslipidemia; ALN: alendronate; ZLN: zoledronate; PRD: prednisolone; MTX: methotrexate; HCQ: hydroxychloroquine; TPTD: teriparatides; ORIF: open reduction with internal fixation; IBG: iliac bone graft; DBM: demineralized bone matrix; mo.: month; LCP: locking compression plate

Patient No.	1	2	3	4	5
Age, years	78	48	53	77	77
Gender	F	F	F	F	F
BMI	21.6	23.3	23.4	28.7	20.9
Symptoms/Trauma	Progressive hip pain, no trauma	Thigh pain, slip and fall	Thigh pain, no trauma	Thigh pain, simple fall	Thigh pain, simple fall
Prodromal pain (duration, months)	3	4	8	1	1
Underlying diseases	RA	SLE, DLP	SLE, DM, HT	SLE, old CVA	DLP
Medication (duration, years)	ALN (7), ZLN (4), PRD (11), MTX (15)	ALN (10), HCQ (14), MTX (2), PRD (2)	ALN (3), PRD (>20)	ALN (14), PRD (15), MTX (15)	ALN (10) ZLN (5)
Femoral stem types (duration prior fracture, years)	Cemented (10.3)	Cemented (15)	Cemented (3)	Cemented (6)	Cemented (10)
Fracture types	Incomplete	Complete	Complete	Complete	Complete
Vancouver classification	B1	B1	B1	B1	B1
Primary treatment	BPs discontinuation, weight-bearing restriction	ORIF with plate and screws + wiring fixation + IBG + DBM + TPTD	Revision femoral long stem + plate augmentation	ORIF with plate and screw + locking attachment plate + TPTD	ORIF with plate and screw + cable fixation + TPTD
Result of 1^st^ treatment	Failure, progressive fracture line	Fixation failure	Union	Fixation failure after re-falling	Union
Union time (months)	-	-	6	-	4
Secondary treatment	Revision femoral long stem + wiring + TPTD (union 8 mo. after operation)	CRIF with revised plate and screws (union 4 mo. after re-operation)	-	ORIF with LCP and IBG with DBM (union3 mo. after re-operation)	-

Case 1

**Figure 1:**
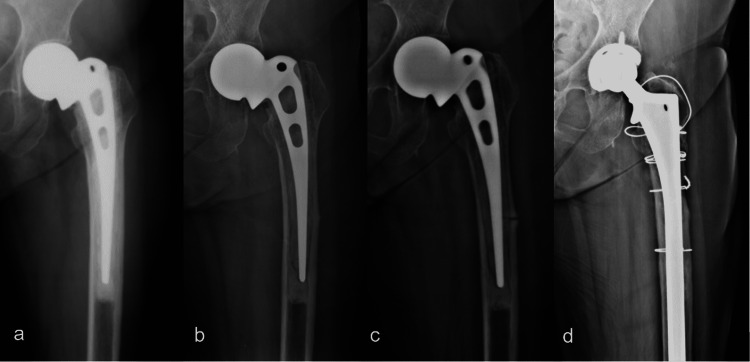
The radiographs from Case 1 (a) Post-operative hemiarthroplasty before APFF occurred, (b) focal lateral cortical thickening with clinical thigh pain at presentation, (c) visible radiolucency line at the lateral cortex thickening, (d) healing of the fracture at eight months after post-operative revision total hip arthroplasty with long femoral stem.

A 78-year-old female presented with left thigh pain for three months in February 2016. She underwent left cemented unipolar hemiarthroplasty for 10.3 years due to a femoral neck fracture (Figure 1a). Her underlying diseases were rheumatoid arthritis (RA) for 15 years and osteoporosis for 13 years. Her medications are shown in Table 1. Radiographs showed focal lateral cortex thickening at mid femoral stem level without visible radiolucency line or definite signs of prosthesis loosening (Figure 1b). She was diagnosed as incomplete APFF and initially treated conservatively by bisphosphonates discontinuation and restricted weight bearing with gait aids. After two months, her pain was reduced, and she was allowed to resume normal activities. However, three months later, she reported progressive hip pain and was unable to walk without a gait aid. The radiographs showed a progressive radiolucency line at the previous cortical thickening (Figure 1c). After discussion regarding the surgical options, revision arthroplasty with a fully coated stem (Solution System, DePuy Synthes, Raynham, MA) and cementless cup (Pinnacle, DePuy Synthes) was chosen due to the risk of progressive acetabular bone loss from the unipolar hip prosthesis. Postoperatively, the patient maintained partial weight ambulation for six weeks and then advanced to full weight-bearing. Teriparatide injection (20 mg/day) was started postoperatively and continued for one year. The incomplete fracture line was obsolete eight months after surgery (Figure 1d). The patient could walk independently without pain and gait aids.

Case 2

**Figure 2:**
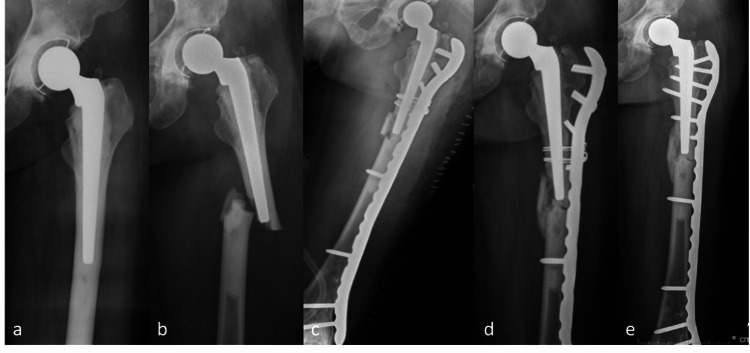
The radiographs from Case 2 a) post-operative total hip arthroplasty before APFF occurred, b) complete fracture originated from the cortical beak with medial spike and minimal comminution, c) immediate post-operative ORIF with plate and screws with wiring, bone graft, and demineralized bone matrix, d) proximal fixation failure with varus malalignment of the fracture at four months after fixation, and e) revision surgery with plate and screw.

A 48-year-old female presented with left PFF after slipping and falling on the floor in January 2017. Previously, she had a history of bilateral osteonecrosis of the femoral heads (ON) and underwent bilateral cemented total hip arthroplasty 15 years prior (Figure 2a). Her underlying diseases were dyslipidemia and systemic lupus erythematosus (SLE) for 18 years. Her medications are shown in Table 1. She reported prodromal thigh pain for four months. Radiographs showed complete transverse fracture with medial spike cracking and a small butterfly fragment at the tip of femoral stem level without signs of prosthesis loosening. She was diagnosed with a complete APFF well-fixed femoral stem (Figure 2b). The fracture was fixed with a 14-hole proximal femoral anatomical LCP splinting the whole femur with unicortical screws and additional wiring fixation at the proximal fragment. Iliac bone graft and demineralized bone matrix (DBM) were added to the fracture gap to stimulate bone healing (Figure 2c). Postoperatively, alendronate was discontinued, and teriparatide injection (20 mg/day) was started. The patient maintained partial weight ambulation with the walker. Technetium (99mTc) bone scan showed negative uptake at the contralateral femur. Four months later, the patient had no pain, and callus formation was detected. However, the unicortical screws were pulled out, and the fracture alignment was changed to varus (Figure 2d). The fixation was then revised with pre-contoured broad-curved LCP without an open fracture site. The fracture achieved union at four months (Figure 2e) after the re-operation, and the patient could walk independently without pain and gait aid.

Case 3

**Figure 3:**
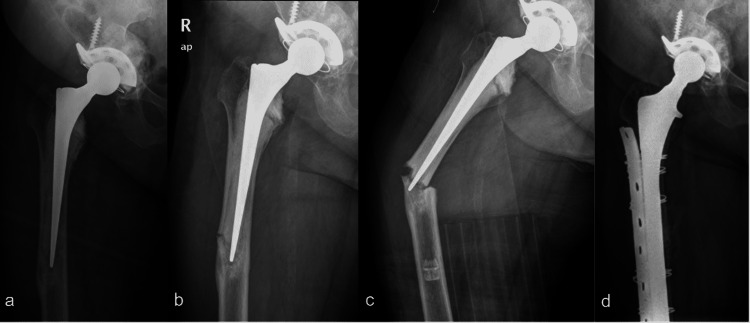
The radiographs from Case 3 a) post-operative total hip arthroplasty before APFF occurred, b) incomplete transverse fracture at lateral cortex with clinical thigh pain at presentation, c) complete fracture progression, d) immediate post-operative revision with long femoral stem augmented with plate and screws with wiring.

A 53-year-old female presented with right thigh pain while walking and a fall in January 2010. Previously, she had bilateral ON and underwent bilateral cemented total hip arthroplasty 18 years prior, followed by revision cemented THR with an anti-protrusion cage due to aseptic loosening (Figure 3a). Her underlying diseases were diabetic mellitus, hypertension, and SLE. Her medications are shown in Table 1. She reported prodromal thigh pain for eight months. Radiographs showed focal lateral cortical thickening with a visible radiolucency line around the tip of femoral stem level without definite signs of prosthesis loosening (Figure 3b). She was initially misdiagnosed with a stress fracture and treated conservatively by weight-bearing protection and vigorous-activity restriction without discontinuation of bisphosphonates. Five months later, a complete fracture developed (Figure 3c). She was therefore diagnosed with a complete APFF with a well-fixed cemented stem. However, due to the previous varus alignment of the stem, revision hip arthroplasty with a fully coated long stem (VerSys Beaded FullCoat Revision Hip Prosthesis, Zimmer Bionet, Warsaw, IN) and augmentation with dynamic compression plate and wiring was chosen (Figure 3d). Postoperatively, the patient maintained partial weight ambulation for 12 weeks and then advanced to full weight-bearing. The fracture was completely healed six months after surgery. She regained the ability to walk without pain and gait aid.

Case 4

**Figure 4:**
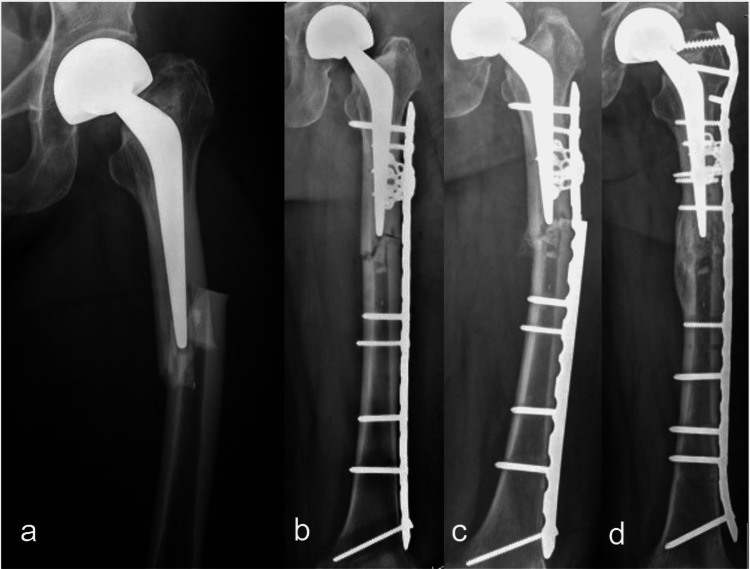
The radiographs from Case 4 a) complete transverse fracture with medial spike with clinical thigh pain at presentation, b) immediate post-operative ORIF with plate and screws with locking attachment plate, c) plate breakage with varus malalignment of the fracture four months after the fixation (clinical pain after re-falling) d) fracture healing consolidation at seven months after the first operation (union at three months after the second operation).

A 77-year-old female presented with left thigh pain after having pre-syncope and falling on the floor in April 2019. Previously, she underwent left cemented bipolar hemiarthroplasty six years prior due to femoral neck fracture and right total hip arthroplasty 11 years prior due to hip osteoarthritis (OA). Her underlying issues were an old cerebrovascular accident with full recovery (old CVA) and SLE for 16 years. Her medications are shown in Table 1. She had prodromal thigh pain for one month. Radiographs showed complete transverse fracture with the medial spike and evidence of focal lateral cortical beak at the tip of femoral stem level (Figure 4a). No signs of prosthesis loosening were detected. The diagnosis was APFF with a well-fixed cemented stem. The fracture was fixed with broad-curved LCP with bicortical and unicortical screws with an additional locking attachment plate at the proximal fragment (Figure 4b). Postoperatively, alendronate was discontinued, and teriparatide injection (20 mg/day) was started. The patient maintained partial weight ambulation with a walker. Follow-up visits showed clinical improvement with progressive callus formation. However, four months later, the patient slipped and fell on the floor again and developed acute pain at the fracture site. Radiographs showed breakage of the plate with intact proximal and distal fixation, and fracture alignment was changed to varus (Figure 4c). The fixation was then revised with pre-contoured broad curve LCP and iliac bone graft and DBM were used to accelerate the healing. The fracture achieved union three months after re-operation (Figure 4d), and the patient could walk independently without pain and gait aid.

Case 5

**Figure 5:**
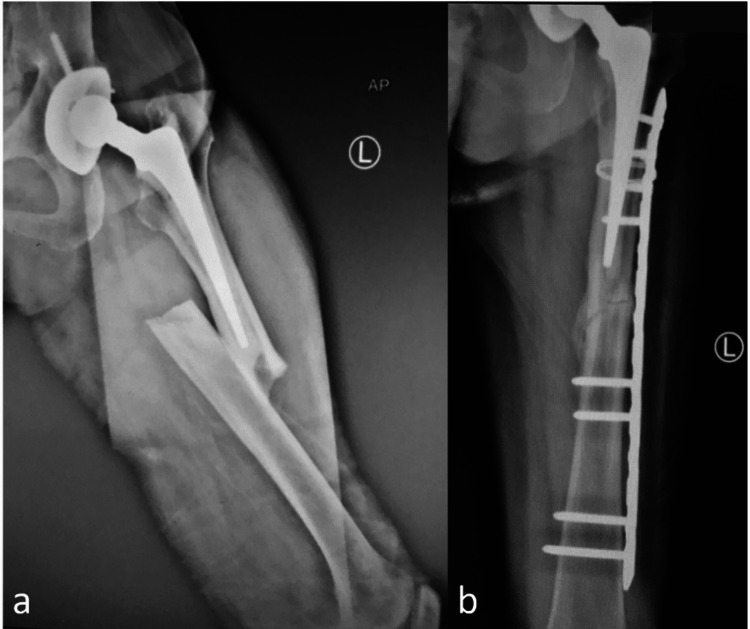
The radiographs from Case 5 a) complete transverse fracture with medial spike with clinical thigh pain at presentation; b) post-operative ORIF with plate and screws with cable wiring four months after fixation.

A 77-year-old female presented with left thigh pain after slipping and falling on the floor in March 2020. Previously, she underwent cemented total hip arthroplasty 10 years prior due to hip OA. Her underlying diseases were dyslipidemia and osteoporosis. Her medications are shown in Table 1. She had prodromal thigh pain for one month. Radiographs showed complete transverse fracture with the medial spike and evidence of focal lateral cortical beak at the tip of femoral stem level without definite signs of prosthesis loosening (Figure 5a). The diagnosis was APFF with a well-fixed cemented stem. The fracture was fixed with broad-curved LCP with bicortical and unicortical screws with additional cable fixation at the proximal fragment. Postoperatively, zoledronic acid was discontinued, and teriparatide injection (20 mg/day) was started. The patient maintained partial weight ambulation for six weeks and then advanced to full weight-bearing. The fracture was completely healed four months after surgery (Figure 5b). She regained the ability to walk without pain and gait aid.

## Discussion

APFFs are fractures characterized by atypical features of AFFs and occur around femoral stem prostheses. Although both APFFs and AFFs would have a theoretically similar pathogenesis, only a few studies have addressed APFF patient characteristics [5], and previous studies showed that APFFs had significantly higher fracture-related complications compared to AFFs [5] or typical PFFs [6,7]. This study aimed to present a case series of five APFF patients and APFF treatment from our experiences. We also reviewed the literature related to the treatment outcome of APFFs and developed an up-to-date treatment strategy. 

Regarding patient characteristics, the findings from this study were mostly comparable to the previous studies that have larger sample sizes [5-8] (Table 2). Our data showed that the APFF patients had common demographic data, such as being female, using long-term bisphosphonates, and having a Vancouver B1 fracture pattern. However, there were differences between this study and prior research, with the current study having a lower mean age (66.6 years vs. 76-80 years), a higher proportion of autoimmune disease (80.0% vs. 41.2%), and a higher proportion of concomitant glucocorticoids or immunosuppressive drug use (80.0% vs. 35.3%) (Table 2). The lower mean age in this study could be explained by the higher proportion of autoimmune disease, resulting in the earlier treatment of glucocorticoid-induced osteoporosis. Moreover, the current results could imply that the patients who had long-term glucocorticoids or immunosuppressive medications also had a higher risk of developing APFFs. Therefore, to achieve the best possible outcome in APFF treatment, the patients underwent hip arthroplasty with risk factors such as older age. Further, patients taking long-term bisphosphonates or glucocorticoids, or immunosuppressive medications should be evaluated for a history of prodromal pain (pain on the thigh, hip, or back during weight-bearing) and carefully checked for abnormal signs on the hip or femur radiographs (localized periosteal thickening with or without transverse fracture line on the lateral cortex between the mid-stem and distal part to the stem). In suspected cases, we recommend using bone scans or MRI for early diagnosis of occult or incomplete APFFs. 

**Table 2:** Comparison between the patients' characteristics in the present study and those in the previous studies with a larger sample size BPs; bisphosphonates, GCs; glucocorticoids *; based on the inclusion criteria of that study

Patients' characteristics	Author, year of publication				
	This study	Robinson et al., 2016 [5]	Mackenzie et al., 2019 [6]	Baba et al., 2021 [7]	De Cicco et al., 2021 [8]
	(n=5)	(n=21)	(n=16)	(n=43)	(n=17)
Mean age, year	66.6	80	73.9	78.0	75.9
Female gender, %	100.0	90.5	87.5	92.3	100.0
Femoral stem design, %					
Cemented	100.0	Mostly cementless	100.0	30.2	35.3
Cementless	0		0	69.8	64.7
Fracture type, %					
Incomplete	20.0	n/a	n/a	14.0	58.8
Complete	80.0			86.0	41.2
Vancouver classification, %					
B1	100.0	100.0	n/a	n/a	70.6
C	0.0	0.0			29.4
BP use, %	100.0	100.0*	81.3	62.8	100.0
Mean BP duration, year	10.6	≥ 2	5.5	6.3	6.3
Autoimmune disease, %	80.0	n/a	n/a	n/a	41.2
GCs or immunosuppressive drugs use, %	80.0	n/a	n/a	n/a	35.3

Concerning the treatment of incomplete APFFs, our findings demonstrated the high failure rate of conservative treatment, which is comparable to the results from a systematic review by De Cicco et al. [8]. In the previous study, the effectiveness of conservative treatment and prophylactic fixation in the incomplete APFFs was 40% (4 in 10) and 83% (5 in 6), respectively [8]. These results were comparable to the results from the systematic review of the treatment of AFFs by Koh et al. as the success rates of conservative treatment and prophylactic fixation in AFFs were 53% and 97%, respectively [4]. The comparable results could imply that both incomplete APFFs and AFFs had a similar prognosis and the same management strategy since discontinuing bisphosphonates, limiting weight-bearing, and giving adequate calcium and vitamin D supplement with or without teriparatide injection should be initiated as soon as the diagnosis was made. Prophylactic fixation should be considered in cases with poor treatment response (such as progression of fracture line or worsening pain or no sign of fracture healing within 2-3 months) or cases with a contralateral previous fracture [8]. 

The result of complete APFFs from the present study showed that there was an implant failure rate as high as 50% (Cases 2 and 4) requiring revision surgery before achieving bone union. Our findings were comparable with those in previous studies since the prevalence of fracture complications requiring reoperation in APFFs varied between 38% and 57% [6,8]. In terms of fixation failure analysis in this study, these cases would be affected by both biological and mechanical factors. In terms of the biological aspect, Cases 2 and 4 would have poor fracture healing due to long-term bisphosphonates therapy and the use of prednisolone and immunosuppressive medications. However, orthobiologic adjuncts (bone graft + DBM + teriparatide in Case 2, and teriparatide in Case 4) were applied for improving fracture healing potential [9], as shown by moderate callus formation at four months (Figure 2) and 3.5 months (Figure 4) postoperatively. Regarding the mechanical aspect, both cases revealed different failure modes-screw back out in Case 2 and broken plate in Case 4. These fixation problems in APFFs were related to the presence of hip prosthesis resulting in difficulty in proximal femur fixation. Therefore, we recommend that the fixation strategy in APFFs be as optimized as possible by applying a long bridging plate with cable or locking attachment plate or secondary plate augmentation for improving proximal femur fixation; using local biological enhancement procedures such as bone grafting, or using DBM; and considering postoperative bone healing stimulation adjuncts, such as teriparatide or low-intensity pulsed ultrasound (LIPUS). 

This study also has some limitations. First, due to the rarity of APFFs and the nature of a retrospective case series, our sample sizes were small, and some risk factors might not be recorded. There was also no comparative group or risk factor analysis. Second, our literature review included only articles from an English-based database and might therefore be inadequate. 

## Conclusions

APFFs are uncommon periprosthetic fractures that share some unique features with AFFs, including patients’ characteristics and fracture-related complications. Early diagnosis and prompt, appropriate treatment are the keys to successful treatment. This study showed that the common patient characteristics in APFFs are being female, using long-term bisphosphonates, and having a Vancouver B1 fracture pattern. Moreover, our results demonstrated a high proportion of patients of younger age having autoimmune disease receiving glucocorticoids and immunosuppressive medications. Therefore, APFF screening should be performed in these high-risk patients, especially on those who had abnormal hip and thigh pain.

Regarding the treatment strategy, APFFs show a poor fracture healing potential and require special attention. Incomplete fracture has a poor-to-fair success rate and mostly requires prophylactic fixation with plate and screws or revision arthroplasty in some cases. Complete fractures require optimization of the fixation strategy and using, if possible, local and systemic biological enhancement for improving treatment outcomes.
